# Supplementary material for: Chromothripsis during telomere crisis is independent of NHEJ, and consistent with a replicative origin
Source: Genome Res. 2019 May;29(5):737–49. doi: 10.1101/gr.240705.118 (PMC6499312; doi:10.1101/gr.240705.118)
Supplement: Supplemental Material [file supp_gr.240705.118_Supplemental_file_1.zip › contigs/annotated_contigs/DB57/contig.2.DB57_length_455_mean_cov_8.61538461538.docx]

**DB57_length_455_mean_cov_8.61538461538**

```
AGGCCTCACTCCCAGCCACTGGACTGCTCCAGCCGACAGAGACAGCCCACGGAGGAAGGAGTGGGGGAAAGGGCTAAGGGCACAAAATG
 >chr7:155154415-155154754 + E=1e-192
TAAAACACGCTCGGGATGCCACCACTTAAAGGGCTAGAGGATGGAGAGAGCAGTGAGGGAGGACGGGCGCTGGGCTGCACACAGCAGGG

GCCCCACCTCACGGGATGCTCTCAGTCTGCAGGCCCAGGGGCTTGTAAGCACCACTGGGGAATTCACTAAACCAGCTTGGAAATTCACT

AAACCAGATTGGAAAAGCCAAGCTCAGGCATAGCCCTCTCCAGCATCCTTGGCTCTCCAGCATCCTCGG|AAA|AAATTTAGTCAGGCA
                                                                      >chr7:135620041-1
TGGTGGTATGCACCTGTAATCCCAGCTACTCGGGAGGCTGAGGCAGGAGAATCGCTTGAACCCAGGAGGCAGAGGTTGCAGTGAGCCGA
35620160 - E=5e-51
GGTTGTGCCACT
```
